# Reducing Avoidable Transfer Delays in the Pediatric Intensive Care Unit for Status Asthmaticus Patients

**DOI:** 10.1097/pq9.0000000000000527

**Published:** 2022-01-21

**Authors:** Takaharu Karube, Theresa Goins, Todd J. Karsies, Samantha W. Gee

**Affiliations:** From the * Department of Pediatrics, Division of Pediatric Critical Care Medicine, Nationwide Children’s Hospital, Columbus, Ohio; † Pediatric Intensive Care Unit Clinical Lead Respiratory Therapist, Nationwide Children’s Hospital, Columbus, Ohio.

## Abstract

**Introduction:**

Status asthmaticus (acute severe asthma) is one of the most common reasons for Pediatric Intensive Care Unit (PICU) admission. Accordingly, ensuring optimal throughput for patients admitted with status asthmaticus is essential for optimizing PICU capacity. Few studies specifically address effective methods to reduce delays related to PICU discharge. This project aimed to identify and reduce avoidable delays in PICU discharge for status asthmaticus patients.

**Methods:**

This quality improvement project focused on reducing transfer delays for status asthmaticus patients admitted to the PICU at a freestanding academic children’s hospital. We standardized the transfer criteria, identified barriers to an efficient transfer, and implemented multidisciplinary interventions. The primary aim was to decrease the average duration from fulfilling the transfer criteria to PICU discharge by 15% from the baseline within 8 months of implementation. The balancing measure was readmissions to the PICU for asthma exacerbations within 24 hours from PICU discharge.

**Results:**

The analysis included 623 patients. Following interventions, the time from fulfilling transfer criteria to PICU discharge decreased from 9.8 hours to 6.8 hours, a 30.6% reduction from baseline. Improvements were sustained for 6 months. In the preintervention group, three patients were readmitted to the PICU within 24 hours of transferring out of the PICU, but no patient was readmitted during the postintervention period.

**Conclusions:**

Standardizing transfer criteria and implementing multidisciplinary strategies can reduce avoidable PICU discharge delays for patients with status asthmaticus. The application of a similar approach could potentially reduce avoidable delays for other conditions in the PICU.

## INTRODUCTION

Asthma is a common chronic condition and is the third leading cause of hospitalization for children nationally.^1,2^ In 2019, the Centers for Disease Control and Prevention (CDC) reported that 5.1 million children in the United States under the age of 18 years had asthma.^3^ Patients with asthma that have progressive respiratory failure resulting in insufficient alveolar gas exchange and need of additional respiratory support, despite using inhaled bronchodilators as first-line therapy, have status asthmaticus or acute severe asthma.^4,5^ As a result, status asthmaticus is one of the most common reasons for admission to the Pediatric Intensive Care Unit (PICU) and can be complicated by high morbidity.^4,6–8^ In response to the high admission rates of these patients, some children’s hospitals have implemented quality improvement (QI) projects to minimize admissions to the PICU and decrease the duration of PICU stay by utilizing clinical scoring systems and pathways to guide therapy.^9–15^ In addition, though not specific to asthma patients, there is growing interest in optimizing hospital-wide patient flow and hospital capacity while maintaining safe and efficient patient outcomes and reasonable hospital staff workload.^16–18^

Prior asthma QI projects at our institution identified transfer delays from the PICU to the inpatient floor services in patients admitted for status asthmaticus. The average duration for the transfer process of these patients, from meeting clinical transfer criteria to physical PICU discharge, accounted for approximately 20% of the total PICU length of stay. Literature describing strategies to reduce avoidable delays in PICU discharge is limited.^19^

This project aimed to reduce avoidable delays in PICU discharge by standardizing transfer criteria and eliminating logistical barriers to promptly transfer patients from the PICU to inpatient floor services for patients with status asthmaticus.

## METHODS

### Setting

This project occurred at Nationwide Children’s Hospital (NCH) in Columbus, Ohio, a freestanding pediatric academic center with 527 beds, including 54 PICU beds. Our PICU often functions near total capacity as there are over 3,000 PICU admissions per year. Pediatric residents and critical care nurse practitioners (NPs) function as frontline providers under critical care fellows and faculty supervision.

At NCH, all status asthmaticus patients, requiring at least one of the following, are admitted to the PICU: fraction of inspired oxygen (FiO_2_) ≥ 0.4, continuous nebulized albuterol, continuous intravenous (IV) infusions related to asthma therapy (eg, magnesium sulfate, beta-agonists, aminophylline, and ketamine), heliox (helium-oxygen mixture), high-flow nasal cannula, noninvasive positive-pressure ventilation, mechanical ventilation, inhaled anesthetics, or extracorporeal membrane oxygenation (ECMO). The respiratory therapists (RTs) utilize an asthma clinical pathway for active patient management and assess patients every 2 hours. Once the patient’s condition improved and met the following standardized criteria, the patient is transfer-ready: FiO_2_ < 0.4, off all continuous IV infusions related to asthma therapy for 4 hours, off high-flow nasal cannula/noninvasive positive-pressure ventilation for 4 hours, and off continuous nebulized albuterol for 4 hours. These patients are primarily transferred to the pulmonology unit or the hospital pediatrics unit with pulmonology consultation depending on staffing and bed availability. PICU practitioners caring for these patients can ultimately hold the transfer based on clinical decision-making.

For this project, we considered a transfer process longer than 4 hours-an avoidable delay based on other studies in the literature.^20,21^ Based on the 16-month preintervention data, the average baseline time of the transfer process was 9.8 hours. The transfer-ready time was determined by adding 4 hours to the time of the last discontinued asthma therapy. The PICU discharge time was defined as the time the patient physically left the PICU, documented in the admission-discharge-transfer notifications in the electronic medical record (EMR). We initiated this QI project to reduce these significant avoidable delays in the PICU transfer process and ameliorate our high PICU occupancy rates. This project was reviewed by the NCH Institutional Review Board and deemed exempt as it met QI project requirements.

### Specific Aims

The project aim was to decrease the average duration of transfer-ready to PICU discharge for status asthmaticus patients by 15%, from a baseline of 9.8 hours to 8.3 hours, within 8 months from the initial intervention and sustain for 6 months. Readmissions to the PICU for asthma exacerbation within 24 hours of PICU discharge served as the balancing measure.

### Interventions

**Fig. 1.**
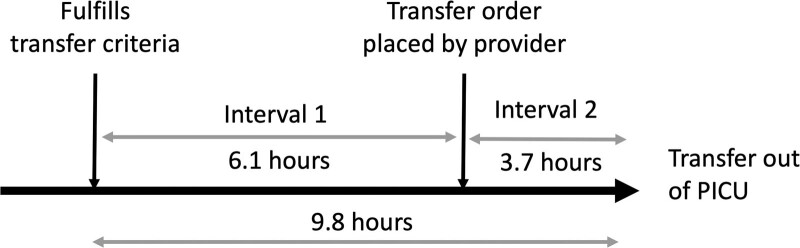
Average timeline and process for patient transfer for status asthmaticus patients.

**Fig. 2.**
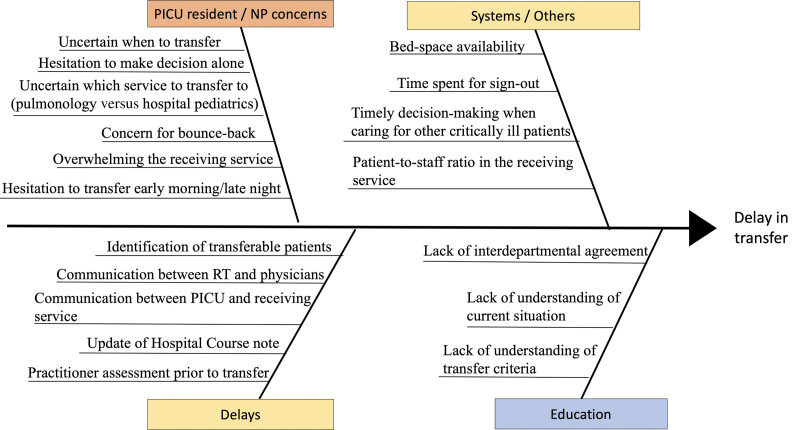
Fishbone diagram depicting potential barriers contributing to avoidable delays in the transfer process.

In November 2018, we formed a multidisciplinary team to work on this QI project, consisting of physicians, PICU RTs and nurses, and data analysts from hospital QI services. The team identified two distinct intervals within the transfer process as opportunities for improvement. Interval 1 is the time from transfer-ready to transfer order placement, and Interval 2 is the time from transfer order placement to PICU discharge (Fig. 1). The team primarily focused on Interval 1, given its longer duration relative to Interval 2, and the ability to immediately intervene with PICU-focused QI interventions. The team interviewed and surveyed PICU fellows, NPs, and residents to identify transfer barriers (Fig. 2—Fishbone Diagram). Based on these results, the team performed plan-do-study-act cycles focusing on two main key drivers, education and communication.

Our team had an interdepartmental meeting to improve communication between services, consisting of physicians, nurses, and RTs from services accepting asthma patients from the PICU, including hospital pediatrics, pulmonary services, and relevant hospital leadership. This meeting in April 2019 focused on removing barriers to timely PICU discharge by applying the transfer criteria without delay and promptly transferring patients, including late night and morning hours. We disseminated consensus agreements to multidisciplinary providers from the PICU, hospital pediatrics, and pulmonary services. Quarterly interdepartmental meetings occurred to review process improvement data and address concerns around patient safety or other improvement barriers.

Starting in May 2019, the team began providing monthly asthma education to residents as they started their PICU rotation. We focused on resident education, as the residents play an essential role but are only in the PICU for a few months throughout their residency and therefore benefit from frequent re-education. We emphasized the importance of frequently communicating with RTs and anticipating when a patient may fulfill the transfer criteria during these monthly didactics. We instructed residents to place transfer orders as soon as patients met transfer criteria, rounding first on the soon-to-be transferred patients and asking to break from rounds to provide sign-out to the receiving service. The cultural change of transferring patients, regardless of the time of day, was also highlighted. We also requested residents prepare parents about the possibility of PICU discharge during the night and morning to prevent transfer delays related to parental hesitation. We reassessed didactics every 3 months, and changes were made based on the feedback. Similarly, we emphasized the transfer criteria to PICU fellows and NPs with posters located in work areas. Emails reminding them about the transfer criteria and updates about the QI project were sent out as regular updates. Lectures were not provided regularly to fellows and NPs, as they are primarily based in the PICU and understand the unit’s workflow. The QI team also had individual conversations when compliance was poor to assess the situation and reiterate the importance of complying with the transfer criteria.

Systems to notify practitioners when a patient was transfer-ready were absent before our interventions. In other words, practitioners were primarily responsible for identifying transfer-ready patients by active surveillance. The team initially considered electronic decision-support Best Practices Alerts in the EMR when patients met transfer criteria. However, due to the complexity of creating an accurate BPA and targeting the correct practitioner for the work shift, in addition to concerns of contributing to pre-existing BPA fatigue, we did not pursue this idea. To promptly identify transfer-ready patients, the team instead requested RTs notify practitioners when a patient fulfilled the transfer criteria, as the RTs assess patients every 2 hours. The notification was mainly by direct verbal communication. To track compliance, we utilized a trackable dot phrase in the EMR to denote the communication occurred. In addition, we asked practitioners to actively identify transfer-ready patients in case the RTs could not communicate promptly.

### Data Collection and Analysis

Data were collected retrospectively for all patients admitted to the PICU with a primary diagnosis of status asthmaticus, age ≥ 2 years, and no pre-existing comorbidity (including congenital heart disease, airway malacia, croup, epiglottitis, bronchiolitis, foreign body aspiration, or allergy to albuterol or ipratropium). We excluded patients with a history of requiring ECMO for status asthmaticus. We collected data by chart review from January 1, 2018, to June 30, 2020. Data from January 1, 2018, to April 30, 2019 were baseline (preintervention), and data from May 1, 2019 to June 30, 2020 were postintervention. Collected data included patient demographics, time of meeting transfer criteria, transfer order placement, and PICU discharge time. Our institution’s QI Services assisted with data acquisition. EMR data were exported into Microsoft Excel spreadsheets (Microsoft, Redmond, Wash.) and imported into SAS dataset (SAS, Cary, N.C.) to create control charts. We created statistical process control charts (X-Bar) depicting the average duration of transfer-ready to PICU discharge and for Interval 1 and 2. We used the American Society of Quality rules to detect shifts in centerline means: 8 consecutive data points, 10 out of 11 data points, or 12 out of 14 data points below average.^22^ Mann-Whitney U or chi-square test was used to compare continuous or categorical variables, respectively. A *P* value of <0.05 was considered statically significant. We performed statistical analyses with GraphPad Prism, version 9.0.0 (GraphPad Software, San Diego, Calif.). We examined data from readmissions to the PICU for asthma exacerbation within 24 hours of leaving the PICU for the balancing measure.

## RESULTS

**Fig. 3.**
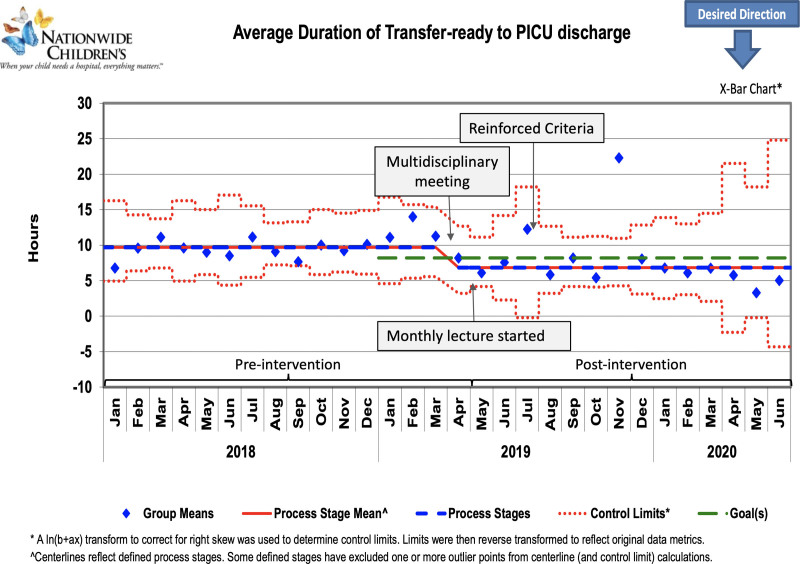
Control chart showing average duration from transfer-ready to PICU discharge. Arrows indicate the timeline of interventions.

**Fig. 4.**
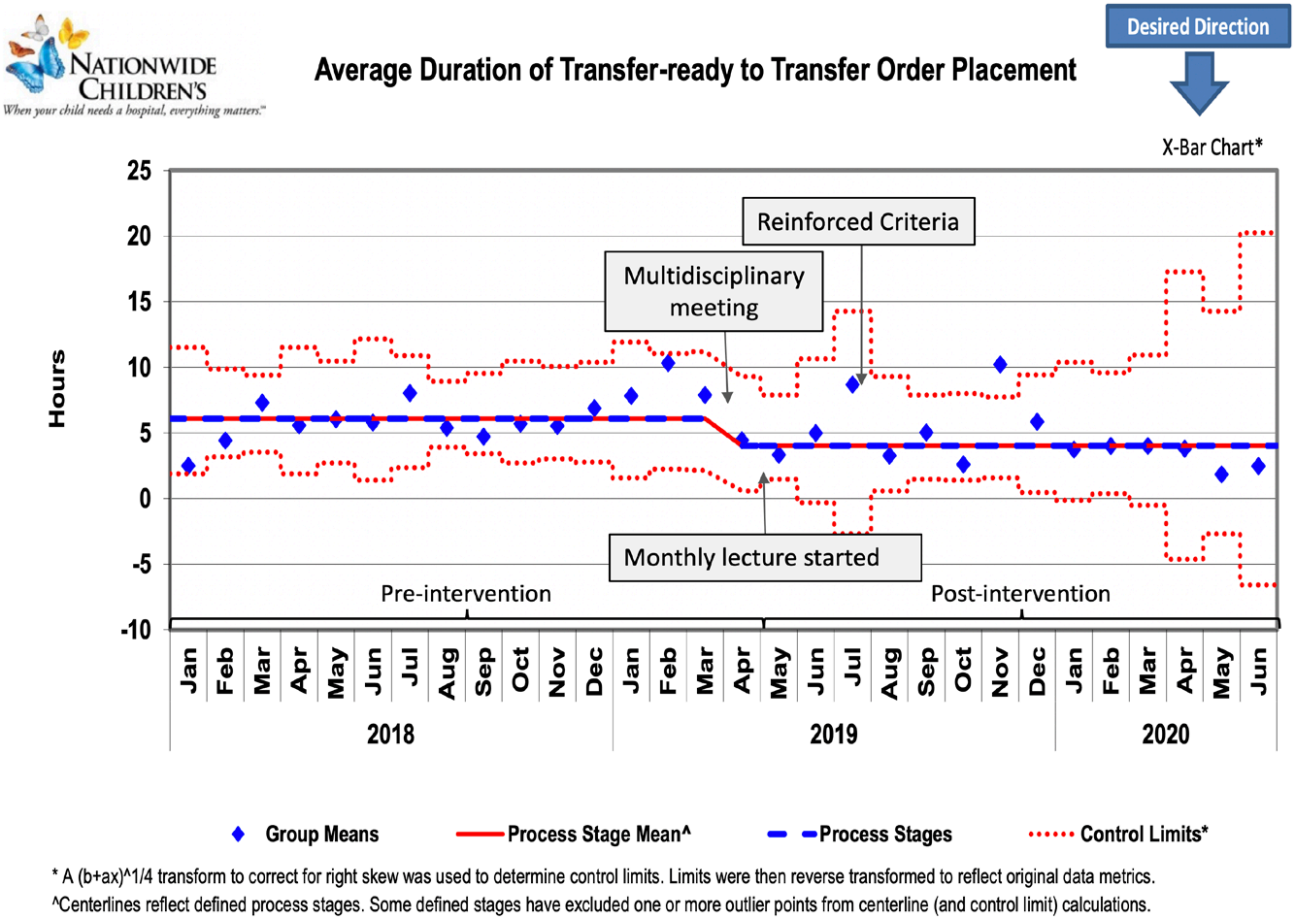
Control chart showing average duration from transfer-ready to transfer order placement (Interval 1). Arrows indicate the timeline of interventions.

**Fig. 5.**
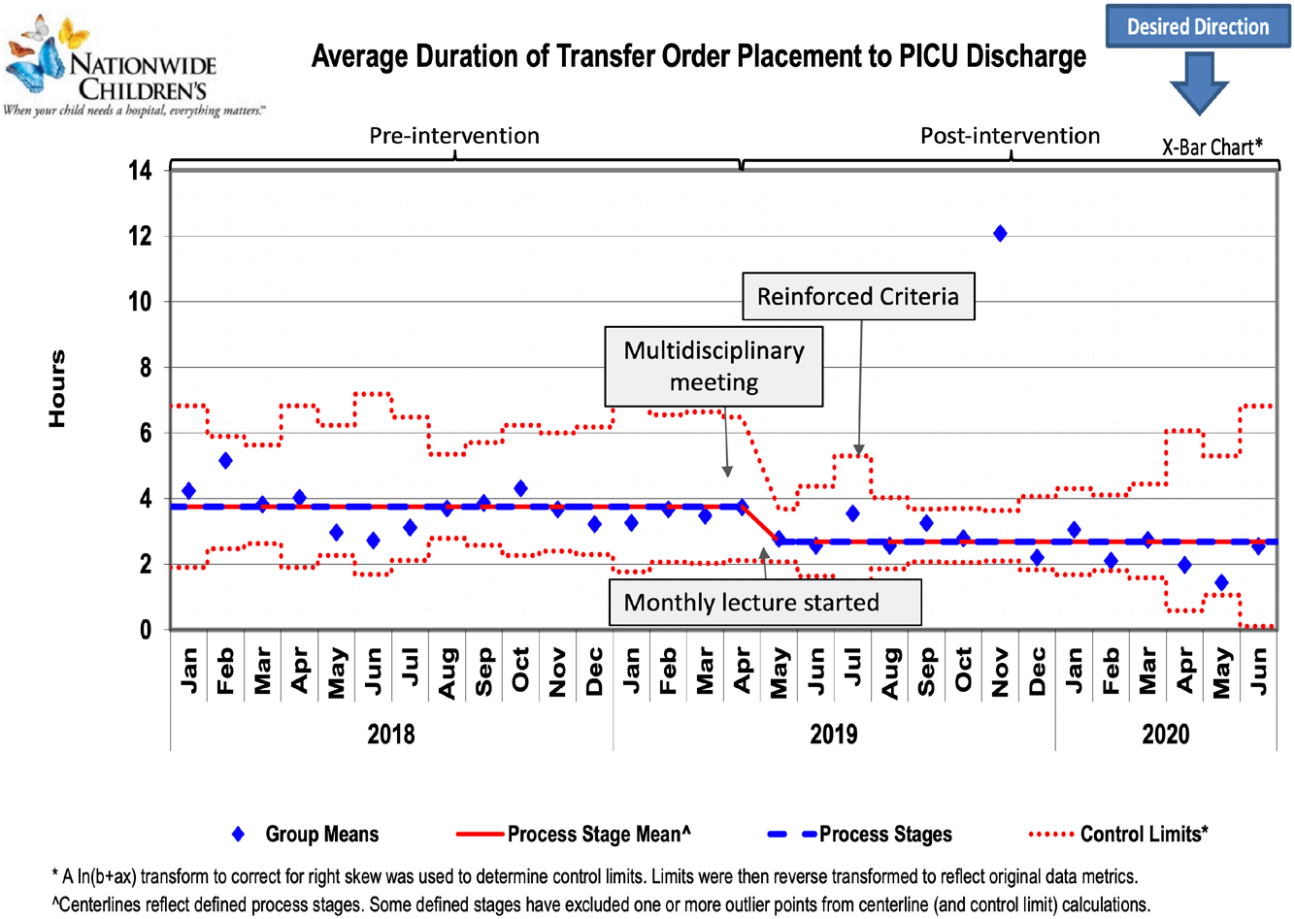
Control chart showing average duration of transfer order placement to PICU discharge (Interval 2). Arrow indicates the timeline of intervention.

A total of 623 patients were included in this project. The pre- and postintervention groups consisted of 389 and 234 patients, respectively. Table 1 depicts comparisons of median age and respiratory support requirements between both groups. As shown in the control charts, there were downward centerline shifts after interventions were implemented (Figs. 3–5). Data from November 2019 was a significant outlier in all three measures. The hospital had an unprecedented number of admissions that led to inadequate bed availability, nursing staff, and RTs, and caused significant delays in patient transfer. This was a special cause variation due to barriers outside this project’s scope; so we did not shift the centerline based on this one outlier.

**Table 1.** Comparison of Age, Respiratory Support, and Distribution of PICU Discharge Time between the Pre- and Postintervention Groups

	Preintervention (n = 389) Median (IQR) or N (%)	Postintervention (n = 234) Median (IQR) or N (%)	*P*
Age (y)	6 (3.0–10.0)	6 (3.0–10.0)	0.99
Respiratory Support			0.25
Noninvasive*	198 (50.9)	107 (45.7)	
Intubation	11 (2.8)	4 (1.7)	
None	180 (46.3)	123 (52.6)	
Discharged Time			0.0006
00:00 to 11:59	58 (14.9)	61 (26.1)	
12:00 to 23:59	331 (85.1)	173 (73.9)	

*Including high-flow nasal cannula and noninvasive positive-pressure ventilation.

IQR, Interquartile range.

The average duration of transfer-ready to PICU discharge decreased by 30.6% from the baseline of 9.8 hours to 6.8 hours (Fig. 3). The subset analysis for the average duration of Interval 1 (Fig. 4) and Interval 2 (Fig. 5) decreased by 2.1 hours and 1 hour, a reduction of 34.4% and 27%, respectively. The goal of decreasing the average duration from transfer-ready to PICU discharge by 15% was sustained for 6 months after December 2019. The distribution of PICU discharge time changed postintervention with an increase in discharge rates from midnight to noon (Table 1). Three patients were readmitted to the PICU in the preintervention group for the balancing measure, but no patients were readmitted in the postintervention group.

## DISCUSSION

We successfully reduced avoidable delays related to PICU discharge for status asthmaticus patients by 30.6% without increasing asthma-related PICU readmissions (balancing measure). Although the average time for the discharge process in the postintervention group was still longer than 4 hours, beyond which we defined as an avoidable delay, we significantly exceeded our initial aim. This project was unique because very few projects focus on reducing delays in PICU discharge as soon as the patient does not require PICU care or resources.

We emphasized practitioner education, interdepartmental collaboration, and communication improvement through iterative plan-do-study-act cycles to address barriers contributing to delays in the transfer process (Fig. 2). The duration of the transfer process showed improvement after the initial interdepartmental meeting. During this meeting, we addressed the transfer criteria and proper transfer of patients despite the time of day. Prospective data sharing among PICU providers led to the earlier placement of transfer orders, particularly during late night and early morning hours, during which time providers were historically hesitant to transfer patients. We believe our focus on resident education significantly contributed to improvement in avoidable delays as they are essential in providing inpatient care. Given their infrequent presence in the PICU, we found frequent refreshers were essential. RTs improved transfer delays by communicating the transfer-ready status of their patients in real-time. Each of these measures led to the earlier identification of transfer-ready patients and timely transfer order placement, thus shortening the duration of the transfer process in aggregate.

Although Interval 2 was not our main focus, there was a 27% improvement. The distribution of the PICU discharge time also changed postintervention with an increase in PICU discharge rates from midnight to noon. These changes are likely multi-factorial but include the collaboration and an overall cultural change of the accepting services. This change of accepting patient transfers during late night to morning hours and the efforts to promptly accept patients when conditions are met speaks to the effectiveness of multiple interdepartmental collaboration.

The clinical significance of a 3-hour reduction in avoidable delays may be equivocal for the patient with asthma. However, this creates additional PICU bed space for 3 hours per asthma patient and improves ICU capacity, and could meaningfully impact other critically ill patients. Several publications indicate that a delay in an ICU admission is associated with worse outcomes.^23–26^ Further, Brennan et al. also commented that a 3-hour reduction in PICU stay per patient could improve patient flow by freeing up beds and improve the efficient admission of acutely ill patients, allowing extra time for providers to offer resources and attention that severely ill patients deserve.^9^

This project has several limitations. First, despite protocolized care, the transfer criteria may not be correct or all-inclusive. As an example, our practitioners reserved the rights to keep patients in the PICU despite meeting the transfer criteria. Elucidating clear practitioner concerns that warrant a delay in transfer was limited by documentation in the chart. Second, this project was dependent on available bed space by the receiving inpatient service. The results were likely affected during the hospital-wide overcrowding through November 2019 to February 2020. This mostly affected the November 2019 data when the hospital had an overwhelming number of admissions, specifically from respiratory illnesses, and suffered from inadequate nursing staff, RTs, and bed availability. Also related to the overcrowding, we acknowledge that, to free up a bed, transfer-ready patients may have been identified much earlier from this impetus and not as a direct result of our QI efforts. Lastly, the number of patients decreased significantly following March 2020, presumably from the statewide coronavirus “stay-at-home” mandates and resultant low hospital census. This may have impacted our project because accepting services were functioning below capacity with ample bed availability. However, the average duration for both Interval 1 and 2 in the postintervention group was the same before April 2020. This suggests that there was no significant impact on our project, but it is difficult to assess the actual effect of this seasonal variability.

For future steps, we plan to streamline further the sign-out process between PICU and accepting providers. Currently, when a patient is transfer-ready, our residents and NPs provide hand-off to the receiving fellow or attending and then subsequently to the receiving resident. This occasionally causes a delay in the transfer process and restricts the PICU providers from performing other tasks as they must wait for the page to be returned. We plan to implement a standardized one-call model, providing hand-off to the receiving fellow or attending who then can share information with their residents. To maintain safe patient care, we initially plan to implement this model for noncomplex patients who only require continuous nebulized albuterol or continuous magnesium sulfate infusion.

## CONCLUSIONS

We successfully reduced avoidable discharge delays for status asthmaticus patients by implementing standardized transfer criteria, stressing early identification of transferable patients, and integrating an interdepartmental, multidisciplinary team approach. To our best knowledge, this project is one of the very few that evaluates and emphasizes the importance of reducing avoidable delays related to PICU discharge. We believe that this concept can be applied to other disease conditions, both medical and surgical. Further studies are needed, exploring the feasibility of defining standardized transfer criteria for other conditions and identifying system barriers to transfer at local institutions with different staffing models. These quality initiatives could meaningfully impact patient flow and improve PICU overcrowding, ultimately improving patient outcomes.
